# The Harveian Oration

**Published:** 1897-10-23

**Authors:** 


### Medical Progress and Hospital Clinics.

*The Editor will be glad to receive offers of co-operation and contributions from members of the profession. All letters should be addressed to* The Editor, at the Office, 28 & 29, Southampton Street, Strand, London, W.C.]

### THE HARVEIAN ORATION.

The Harveian Oration, which was delivered before the Royal College of Physicians, on Monday, by Sir William Roberts, M.D., F.R.S., was a graceful and scholarly address. He devoted himself principally to drawing a contrast between the limits which are naturally imposed on progress in literature and in art, and the lack of all limits to progress in science, and he led up in a striking and convincing manner to his deduction that, although the history of civilisation had been largely a history of the rise and fall of one form of society after another, the form of civilisation which was now spreading over the globe, instead of falling, would endure, in that it was founded upon science, on whose progress no limitation could be placed.

After sketching the main points in Harvey's career, and stating that this year was the 300th anniversary of his graduation at Cambridge in 1597, he pointed out that Harvey's lifetime marked the commencement of a new era in human progress—the era of exact science—which in the present age was slowly but surely transfiguring the aspects and prospects of civilised society.

All the older civilisations, he said, have issued either in extinction or in stagnation. Those of Egypt and Chaldæa, of Greece and Rome, after a phase of progressive decline, have eventually perished by military conquest. The ancient civilisations of the Far East—those of India and China—still persist, and have a semblance of life; but it is a life of helpless torpor and immobility. Is our modern civilisation doomed to share a kindred fate? There are, I think, good reasons for believing that in this respect history will not repeat itself. It may be said broadly that the older civilisations rested essentially upon art and literature (including philosophy), and that modern civilisation rests, in addition, upon science and all that science brings in its train. This difference is, I think, fundamental, and connotes a radical difference as regards stability and continuance between ancient and modern society.

It is both striking and interesting to observe how again and again literature and the fine arts have seemed to reached an acme, a point of perfection beyond which it would appear impossible for them to go, and have then declined. Probably the explanation is that these arts are but the expressions of man's own emotions, and that the organisation of man himself opposes limits to the possible development of his arts and his literature. These, in fact, are subjective, while science is objective, and by the invention of new instruments and the discovery of new substances is constantly, as it grows, extending for itself the field over which it can develop and expand.

The ancients had a vast store of knowledge that was of inestimable value, but it was not science in the modern sense of the word. It was not systematised and interpreted by co-ordinating principles, nor illuminated by generalisation which might serve as guides to further acquisitions. It grew by casual additions, and was liable at any time to be swept away into oblivion by the flood of barbaric conquest.

It is quite obvious that there came into the world of natural knowledge about the time of Galileo and Harvey a something—a movement, an impulse, a spirit—which was distinctly new, which Bacon, with prophetic insight, termed "a new birth of time."

There arose a distrust of the dicta of authority and an increasing reliance on ascertained facts, which came to be regarded as the only data upon which natural knowledge could be securely founded. Experiment came into vogue, instruments were invented, and discoveries were no longer stumbled upon, but were gathered in as the fruit of systematic and purposive research.

Thus the sciences advanced and are still advancing, step by step, by mutual help at an ever increasing speed, pushed on by that irresistible forward impulse which has characterised the scientific movement from its inception. This movement has now become the dominant factor in civilisation. There is no doubt that under the reign of science a striking amelioration in the state of society has taken place, and this is not confined to the material and physical well-being of the population. There is some evidence that the complex of condition which we term "modern civilisation" is acting favourably in the direction of making people more reasonable and better conducted, and the whole environment of modern life seems in several ways calculated to foster habits of correct thinking and acting.

But, say the prophets of evil, "this will not endure; modern civilisation based on science will in time go the way of all its predecessors, and end in extinction or in decay and stagnation." It is proverbially unsafe to dogmatise about the future; this, however, may be affirmed—that, if modern civilisation is to come to an end, it will not perish in the same way nor from the same causes as previous civilisations. One of the standing perils of civilised communities in ancient times was the risk of being overwhelmed by hordes of barbarian invaders. This danger no longer threatens us. Power has passed for ever into the hands of nations which cultivate science and invent, for the issue of battle is now decided in the laboratories of the engineer and chemist. A civilisation founded on science must endure, in that the scope of science knows no limit.

